# Response selection difficulty modulates the behavioral impact of rapidly learnt action effects

**DOI:** 10.3389/fpsyg.2014.01382

**Published:** 2014-12-15

**Authors:** Uta Wolfensteller, Hannes Ruge

**Affiliations:** ^1^Department of Psychology, Technische Universität DresdenDresden, Germany; ^2^Neuroimaging Center, Technische Universität DresdenDresden, Germany

**Keywords:** ideomotor theory, learning, goal-directed behavior, differential outcomes, action effects, rule learning, S–R mapping, compatibility

## Abstract

It is well-established that we can pick up action effect associations when acting in a free-choice intentional mode. However, it is less clear whether and when action effect associations are learnt and actually affect behavior if we are acting in a forced-choice mode, applying a specific stimulus–response (S–R) rule. In the present study, we investigated whether response selection difficulty imposed by S–R rules influences the initial rapid learning and the behavioral expression of previously learnt but weakly practiced action effect associations when those are re-activated by effect exposure. Experiment 1 showed that the rapid learning of action effect associations is not directly influenced by response selection difficulty. By contrast, the behavioral expression of re-activated action effect associations is prevented when actions are directly activated by highly over-learnt response cues and thus response selection difficulty is low. However, all three experiments showed that if response selection difficulty is sufficiently high during re-activation, the same action effect associations do influence behavior. Experiments 2 and 3 revealed that the effect of response selection difficulty cannot be fully reduced to giving action effects more time to prime an action, but seems to reflect competition during response selection. Finally, the present data suggest that when multiple novel rules are rapidly learnt in succession, which requires a lot of flexibility, action effect associations continue to influence behavior only if response selection difficulty is sufficiently high. Thus, response selection difficulty might modulate the impact of experiencing multiple learning episodes on action effect expression and learning, possibly via inducing different strategies.

## INTRODUCTION

One prerequisite for voluntary, goal-directed action is the ability to form associations between actions and their consequences. More importantly even, we also need to take into account the situational context, as waving and shouting might be the action to choose to get me a drink in a crowded pub, while instead it might get me thrown out of a fancy restaurant. Thus, adaptive goal-directed actions require the rapid extraction of the relation between stimulus situation (S), potential responses (R), and to be achieved effects (E). It is well-established that we can pick up action effect associations while performing freely chosen actions (Elsner and Hommel, 2001; Herwig et al., 2007; Herwig and Waszak, 2009; Herwig and Horstmann, 2011; Ziessler et al., 2012). However, results are less unequivocal with respect to situations when actions are chosen according to a stimulus–response (S–R) rule.

The acquisition and expression of action effect associations have been extensively studied with variants of the classic two-phase action effect induction paradigm (Greenwald, 1970; Hommel, 1996; Elsner and Hommel, 2001). This paradigm encompasses an acquisition phase in which participants’ responses are followed by specific auditory or visual effects. In a subsequent test phase, participants respond to these effects in a manner that is either compatible or incompatible with action effects learnt in the preceding acquisition phase. Following the logic of the Ideomotor Theory (Lotze, 1852; Harless, 1861; James, 1890; Hommel et al., 2001) bidirectional associations are formed between responses and effects. Therefore, incompatible responses are expected to be slower or less frequent than compatible ones because perceiving the effect would reactivate the learnt response-effect association and hence prime the response that previously caused that effect. Indeed, this pattern of results was observed in numerous experiments (e.g., Elsner and Hommel, 2001; Herwig et al., 2007; Herwig and Waszak, 2009; Pfister et al., 2011; Wolfensteller and Ruge, 2011). Yet, it is still debated under which circumstances action effect relations are learnt and actually influence behavior.

One of the most hotly debated questions is whether learning and expression of action effects are restricted to a so-called ‘intentional action mode,’ investigated via free-choice actions, were participants choose a left or right button presses as they like. In some studies, response-effect compatibility did not influence behavior in a so-called ‘stimulus-based action mode,’ when responses had been forced-choice, based on an S–R rule such as for instance pressing the left button upon seeing a leftward pointing arrow, but a right button upon seeing a rightward pointing arrow, during learning (Herwig et al., 2007; Herwig and Waszak, 2009; Herwig and Horstmann, 2011). Based on that it was proposed that ideomotor action effect learning was constrained to cases were people freely choose an action rather than respond to a stimulus (Herwig et al., 2007). However, several other studies have reported an influence of action effect compatibility on free-choice behavior at test irrespective of the action mode of the preceding learning phase (Dutzi and Hommel, 2009; Pfister et al., 2011; Janczyk et al., 2012). From that we can conclude that a free-choice action mode is not a prerequisite for action effect learning.

Accordingly, it was proposed that instead the behavioral expression of these learnt action effect associations depended on a free-choice action mode (Pfister et al., 2011). However, using a related experimental approach capitalizing on pre-existing response-effect compatibility (Kunde, 2001), it has been shown that action effects can influence responses based on an S–R rule if the response to the stimulus was chosen in order to achieve a certain effect (Ansorge, 2002; Pfister and Kunde, 2013; Zwosta et al., 2013; Pfister et al., 2014b). Moreover, several studies using the two-phase action effect induction paradigm have also reported evidence for the behavioral expression of action effect associations while responding to a stimulus according to an S–R rule in the test phase without being explicitly instructed to produce a certain effect (Elsner and Hommel, 2001; Experiment 1; Ziessler and Nattkemper, 2002; Ziessler et al., 2004; Wolfensteller and Ruge, 2011). From that we can conclude than that a free-choice action mode is not a prerequisite for the expression of action effects either.

Still the puzzle why S–R rule-based behavior is affected by action effect associations in some studies but not in others, remains. Notably, a quite similar pattern of mixed results emerged with respect to the differential outcome effect in conditional S–R learning (Trapold, 1970). If actions are followed by differential outcomes, i.e., specific action effects, rather than a common reward outcome, S–R learning is reliably faster, and more accurate in animals (Trapold, 1970). In humans, however, the differential outcome effect seems to depend on the difficulty of the task at hand, emerging only for more difficult choice problems, involving for instance more than two items (Estevez et al., 2001; Plaza et al., 2011) or arbitrary rather than only spatial cues (Legge and Spetch, 2009). More precisely, it seems to depend on whether the choice problem is sufficiently complex for the individual under investigation. Thus while choosing among two alternatives is sufficiently complex for 5-year olds to benefit from differential outcomes, 7-year olds do benefit only when choosing among four alternatives (Estevez et al., 2001). Analogously, Mok and Overmier (2007) reported that choice problems with less than four choice options were too easy to produce a differential outcome effect in healthy adults. However, when learning to classify more hard-to-discriminate stimuli, performance is boosted by differential outcomes. In line with that, Gaschler and Nattkemper (2012) recently showed that if stimulus discrimination is challenging, but effects are salient, action effect associations influence response speed irrespective of the action mode. In contrast, if the effects were not salient and thus not very efficient as additional discriminative cues, their impact on behavior was substantially reduced and did not emerge until after substantial amount of practice, if at all.

If we consider previous studies in this regard, it turns out that action effect learning or expression is typically reported in studies using rather complex stimulus-response-effect (S-R-E) mappings. For instance, Wolfensteller and Ruge (2011) used a mapping linking four visual stimuli with two button presses and four color effects while Ziessler and Nattkemper (2002) used an even more complex mapping linking eight numbers and letters via four button presses to eight different numbers and letters in both acquisition and test phases. In comparison, studies using rather simple S-R-E mappings typically report no evidence for action effect learning or expression. For instance, Herwig et al. (2007) and Herwig and Waszak (2009) used mappings linking only two stimuli to two responses and two effect stimuli in the acquisition phase and two auditory effects with two responses in the test phase. The number of choice alternatives is known to be one factor determining response selection efficiency (Sanders, 1998; Proctor and Vu, 2006). In that sense, response selection in the studies that did not find evidence for action effect learning when responding according to an S–R rule was apparently much easier.

However, reducing the number of choice options is only one way to make response selection easy. Response selection demands were further lowered in studies using spatial stimuli (Experiment 1 and Experiment 3B in Herwig et al., 2007; Pfister et al., 2010; Herwig and Horstmann, 2011). Spatial stimuli directly and automatically activate the corresponding response due to the spatial overlap of stimulus and response, i.e., due to long-term associations (Kornblum et al., 1990). This automatic response activation results in the well-known behavioral benefit of spatial S–R compatibility (Fitts and Seeger, 1953; Fitts and Deininger, 1954). In contrast, stimuli lacking dimensional overlap with the response rely on the indirect, more controlled route of response activation requiring active response retrieval (an S–R table search, Kornblum et al., 1990) based on instructed S–R rules, i.e., short-term associations. Obviously, actively retrieving a response renders response selection more difficult than if the response was directly activated. In line with that, action effects can be learned also while responding according to simple S–R rules comprising only two stimuli and two responses, as long as direct response activation is prevented (Hommel, 1996). Thus when responding to a stimulus (or former action effect) according to a rule, this rule might need to pose sufficient demands on response selection to enter an action mode in which action effect associations can actually affect behavior.

The present study set out to test the influence of response selection difficulty on the rapid learning and the expression of action effect associations. To this end, we conducted three experiments using a novel version of the aforementioned two-stage action effect induction paradigm (Greenwald, 1970; Elsner and Hommel, 2001) adapted for investigating rapid action effect learning (Wolfensteller and Ruge, 2011; Ruge et al., 2012). In this rapid action effect learning paradigm, participants perform multiple brief acquisition and test phases, one for each novel S-R-E mappings. Each of these novel mappings links four unique visual stimuli to four manual button presses and four unique sound effects. Learning of action effect associations is immediately probed in the test phase, when participants have to respond to the former action effects by button-press. For each mapping, the responses are compatible with the previously acquired action effect association for two effects, and incompatible for the two other effects. The within-subject compatibility effects in accuracy and response times indicative of action effect learning have previously been shown to emerge already after eight repetitions of each S-R-E pair (Wolfensteller and Ruge, 2011). Crucially, in the present study, we manipulated response selection difficulty in two ways. First, response cues (RCs) were presented for a different number of repetitions of each stimulus or effect in the learning phase (Experiment 1) and test phase (all experiments) to instruct novel stimulus-to-response mappings and effect-to-response mappings, respectively. Second, response selection difficulty was manipulated by providing different types of RCs (Experiments 1 and 2). Of note, one of the fundamental differences between previous studies on action effect learning and the rapid action effect learning paradigm is the experience of multiple learning episodes, instead of just one long one, which might also contribute to the discrepant findings. We therefore additionally explored the impact of multiple learning episodes on action effect learning and expression and its relation to response selection difficulty. Finally, response selection difficulty generally increases response times, and the impact of response-effect compatibility has been shown be more pronounced at the faster tail of the response time distribution (Hommel, 1996; Kunde, 2001; Paelecke and Kunde, 2007; Pfister et al., 2014a). Therefore we conducted a third experiment to test whether the effects of response selection difficulty can be alternatively explained by simply giving effects more time to prime associated actions (Paelecke and Kunde, 2007).

## EXPERIMENT 1

In Experiment 1, participants’ responses to arbitrary visual stimuli in the learning phase were instructed and guided by additional spatially compatible RCs. Response selection difficulty during learning was manipulated between-participants, by providing these RCs for a differing number of trials. If all trials are spatially guided, the difficulty and need for response selection is low, because spatially compatible RCs would automatically activate the correct response, without the need to ever retrieve the correct S–R rule-based on the actual stimulus alone. If response selection difficulty affects action effect learning, then the compatibility effect in the test phase should be larger when less RCs were provided in the learning phase. Response selection difficulty during test was manipulated by presenting trials with and without RCs in both groups. If the behavioral expression of learnt R-E associations depends on response selection difficulty, the compatibility effect should increase in test trials without additional RCs.

### MATERIALS AND METHODS

#### Participants

Fifty young adults participated in the experiment (21 male, mean age 26.3 years) and received monetary compensation (5 20AC) or course credit. Participants were divided into two groups of 25 participants each^1^. All reported experiments were conducted in accordance with the Declaration of Helsinki and the ethical guidelines of the German Association of Psychologists (DGPs). Formal approval of the local ethics committee was not required as no risk of deception of the participants was involved. Written informed consent was obtained from all participants prior to the experiment.

#### Stimuli and procedure

**FIGURE 1:**
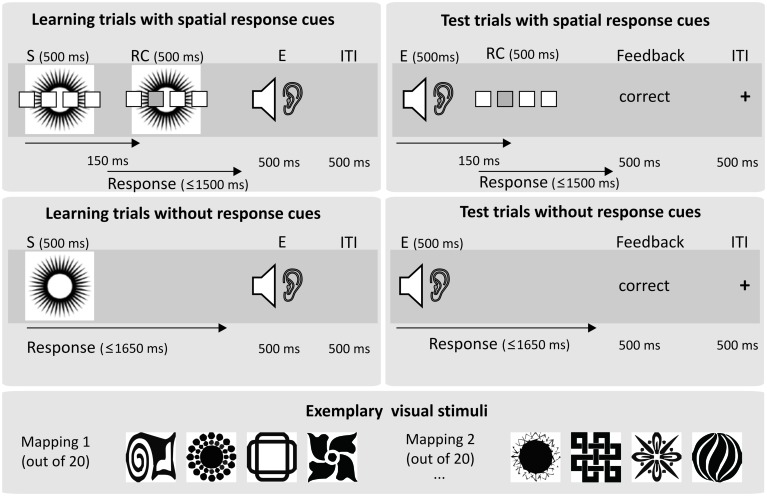
**Basic experimental paradigm.** Each experimental block comprised a learning phase and a test phase. In the learning phase, participants learned to respond to one out of four visual stimuli (S) by pressing one out of four buttons. Groups differed in respect to the amount or type of response cues (RCs) given in addition. In the fully guided spatial group, a spatial response cue highlighting the location of the correct response was presented in all learning trials. In the partly guided spatial group, spatial response cues were presented only for the first three trials for each stimulus. In the remaining 24 learning trials the response cue was not informative (four empty boxes). After a correct response, one out of four sounds was presented as an action effect (E). The test phase was the same for all groups. Participants had to respond to the sound stimuli which had served as action effects in the learning phase. Response cues indicating the correct responses were again presented for the first three repetitions of each former action effect. In the remaining 24 test trials, no response cues were presented. Visual feedback concerning the correctness of the response was given on each trial. In Experiment 2, letters were presented as response cues in learning and test trials. In Experiment 3, the onset delay between effect tones and spatial response cues was increased to 450 ms in the test phase.

A detailed description of the trial timing is given in **Figure 1**. The experiment comprised 20 experimental blocks each consisting of a learning phase and a test phase for a novel 4-4-4 S-R-E mapping (see **Figure 1**). Participants were instructed that in the first part of each experimental block they would learn to respond to four visual stimuli by pressing one out of four buttons and that correct responses would be indicated by four sounds. Furthermore, they knew that they would have to learn how to respond to the sounds in the second part of each block. Eighty-four unique black and white images were used as visual stimuli (http://www.mattonimages.de/bilder/cd/ingram_publishing/creative_symbol_collection/), and eighty-four unique natural sounds (such as e.g., dog bark) served as sound effects. Stimuli and sounds were pre-arranged in sets of four. Those sets were randomly assigned to the blocks, resulting in a different assignment of stimulus sets to blocks in all participants. Importantly, the correct response to a particular stimulus was contingently followed by a specific sound in each block. Visual stimuli were presented on the computer monitor in a distance of ∼60 cm to the subjects’ eyes. Responses were delivered by left and right index or middle finger presses using keyboard buttons D, F, K, and L. The sounds were presented via headphones. The experiment was controlled via EPrime 2.0 running on a standard PC.

For one group of participants, response selection during learning was instructed and guided by a spatial RC presented 150 ms after stimulus onset for all eight repetitions of each stimulus (*fully guided spatial, N = 25*). Another group of participants received this spatial guidance only for the first three repetitions of each stimulus (*partly guided spatial, N = 25*). Participants in the fully guided group knew that the RCs would be provided throughout the learning phase, and participants in the partly guided group knew that the spatial RCs would occur only in the initial phase, but that they had to retrieve the correct response to each stimulus from memory after that. Apart from the RCs, no other instructions about the currently valid S–R rules were given. Consequently, for the fully guided group, response selection was very easy as they could in principle rely on direct response activation due to the spatial correspondence between the RC and the required response for all trials. In contrast, for the partly guided group, response selection was more difficult (involving active retrieval) at least during the unguided learning trials because they could only use the arbitrary visual stimuli that bore no spatial correspondence with the response. In both groups, correct responses were immediately followed by one of four auditory effects, erroneous responses or misses led to a visual error feedback and the trial was immediately repeated. After 32 correctly answered trials the learning phase ended. Thus, it was ensured that participants always experienced exactly eight repetitions for each response-effect pair.

In the subsequent test phase, participants had to respond to the former action effects. Crucially, the assignment of two former action effects to responses was compatible and the assignment of the other two action effects was incompatible with the action effect associations acquired in the learning phase. Incompatibility was implemented such that participants had to either respond with the other finger of the same hand, or with the same finger of the other hand. The resulting four pairings of compatibility, fingers and hands were balanced across blocks. For both groups, response selection for the first three presentations of each former action effect was again instructed by spatial RCs (three correct trials per effect), while for the remainder of the test phase (six trials per effect) no RCs were presented. Thus, participants could rely on direct response activation in test trials with RCs due to the spatial correspondence of RC and response. Compared to that, response selection was more difficult in test trials without RCs, because the correct response to the former action effects had to be retrieved from memory. After the last test trial, the next learning phase for a new S-R-E mapping started.

### RESULTS

We excluded the very first learning trials (3.1%) and test trials (2.8%) for each mapping to exclude possible unspecific effects of switching from learning to test phase or vice versa, i.e., having to respond to auditory rather than visual stimuli. Accuracy and mean reaction times (RTs) of correct responses were calculated separately for each stimulus repetition in the learning phase and for each stimulus repetition of compatible and incompatible trials in the test phase. For consistency, RT was calculated in reference to the onset of the visual or auditory stimulus for all trials.

#### Learning of action effects

The main focus of the present study is on the test phase, but we include an analysis of learning phase data to confirm that the RCs were of instructional value to the participants. RT and accuracy were entered into separate repeated measures analysis of variances (ANOVAs) involving the eight-level within-subject factor *stimulus repetition* and the between-subject factor *response selection difficulty* during learning (fully vs. partly guided spatial group).

Unsurprisingly, RT decreased from 719 to 622 ms over the course of the learning phase, *F*(2.1,103.9) = 43.01, MSE = 3584.80, *p* < 0.001, $\eta_{p}^{2}$ = 0.47. Furthermore, there was a significant interaction of stimulus repetition and response selection difficulty during learning *F*(2.1,103.9) = 16.39, MSE = 3584.80, *p* < 0.001, $\eta_{p}^{2}$ = 0.25. *Post hoc t*-tests indicated that the two groups were indistinguishable in trials with RCs, all *t* < 1.2, *p* > 0.05 (Bonferroni-corrected). However, without RCs, participants in the partly guided group responded faster than the ones in the fully guided group. This speed-up increased up to 56 ms at stimulus repetition 8, *t*(48) = 2.92, *p* < 0.05 (Bonferroni-corrected), but was numerically already present for the last stimulus repetition with RCs (21 ms). In fact, when RCs were presented, the decrease in RT from stimulus repetition 1–3 was significantly steeper in the partly guided group (95 ms) compared to the fully guided group who received RCs throughout the whole learning phase [15.7 ms, *t*(30.27) = 4.61, *p* < 0.001]. This indicates that the partly guided group started using the S–R associations already more than the fully guided group even though the first three stimulus repetitions were physically identical in both groups.

For accuracy, a significant main effect of stimulus repetition, *F*(2.65,127.2) = 23.29, MSE = 20.25, *p* < 0.001, $\eta_{p}^{2}$ = 0.33, was further qualified by a significant interaction of stimulus repetition and response selection difficulty during learning, *F*(2.65,127.2) = 26.25, MSE = 20.25, *p* < 0.001, $\eta_{p}^{2}$ = 0.35. Most importantly, there was a moderate temporary dip in accuracy from stimulus repetition 3–4 in the partly guided group [98.1 vs. 88.0%, *t*(24) = 7.14, *p* < 0.001] which was not present in the fully guided group [98.8 vs. 99.6%, *t*(24) = –1.24, *p* > 0.20]. Again, this pattern of results is exactly what would be expected if participants had successfully used the RCs as instruction but had also started learning the S–R associations during the first three stimulus repetitions.

#### Behavioral expression of action effects

For the test phase, RT and accuracy measures were averaged separately for trials with and without RCs, i.e., stimulus repetition levels 1–3, and 4–8 respectively. This data was subjected to repeated measures ANOVAs including within-subject factors compatibility (of the effect-response mapping) and response selection difficulty at test (trials with and without RCs), and between-subject factor response selection difficulty during learning (fully vs. partly guided spatial group).

**FIGURE 2:**
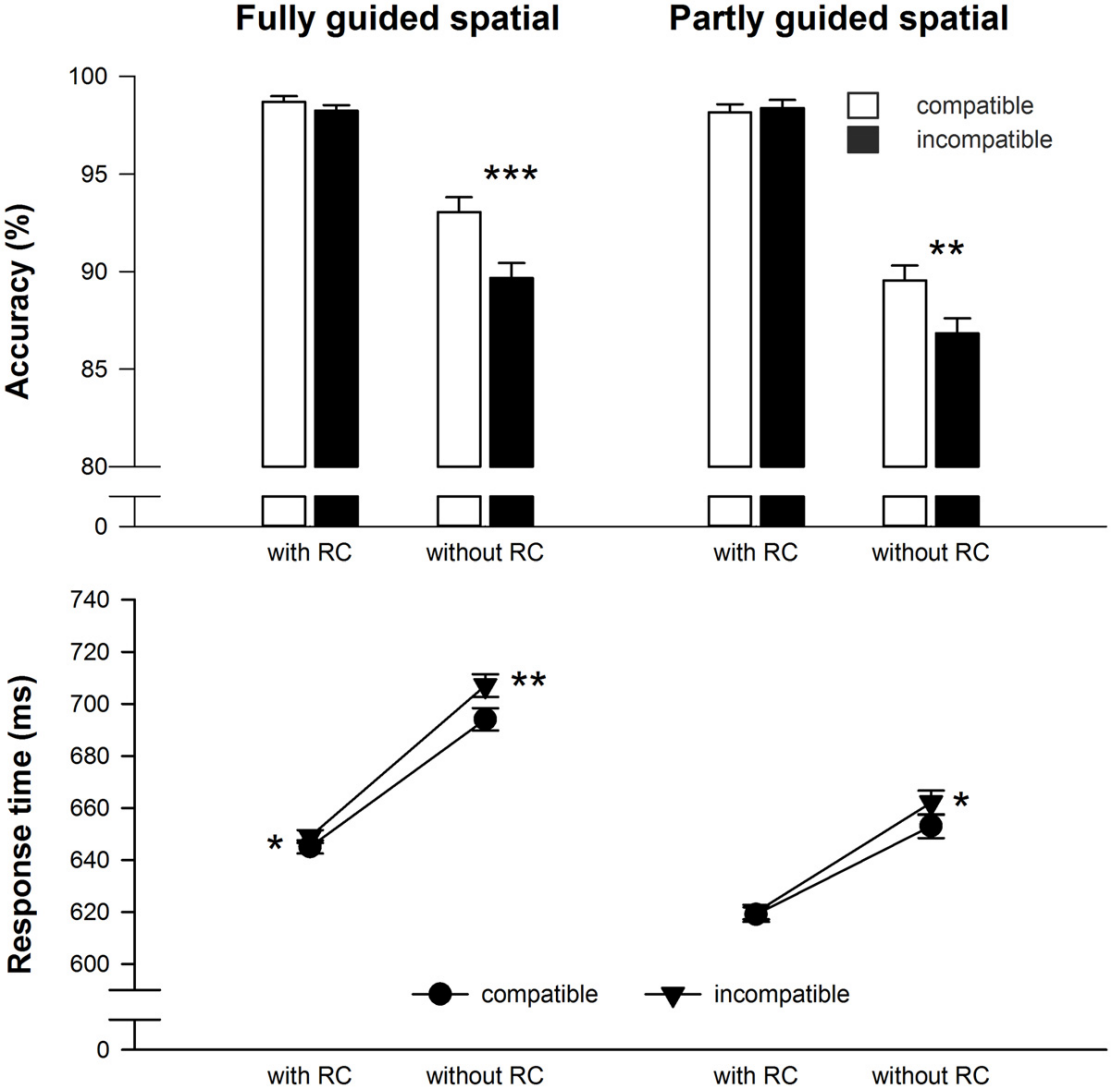
**Mean reaction time and accuracy data for the test phase in Experiment 1.** Error bars denote SE_PD_, the standard error of the mean paired (within-subject) difference between two conditions (Pfister and Janczyk, 2013). Asterisks denote significantly longer or less accurate responses for incompatible compared to compatible effect-response mappings, *p < 0.05, **p < 0.01, ***p < 0.001, all one-sided paired *t*-tests.

For RT, main effects of compatibility, *F*(1,48) = 13.1, MSE = 179.34, *p* < 0.01, $\eta_{p}^{2}$ = 0.21, and response selection difficulty during test *F*(1,48) = 35.0, MSE = 2969.46, *p* < 0.001, $\eta_{p}^{2}$ = 0.42, emerged, which were qualified by a two-way interaction (see **Figure 2**), *F*(1,48) = 5.0, MSE = 158.30, *p* < 0.05, $\eta_{p}^{2}$ = 0.09. Participants responded significantly faster in trials with RCs (634 ms) compared to trials without RCs (679 ms) illustrating the difference in response selection difficulty during the test phase. Furthermore, responses in compatible trials were faster (653 ms) than in incompatible trials (660 ms). Importantly, paired *t*-tests revealed reliable compatibility effects for the test trials without additional RCs only (see **Table 1** and **Figure 2**). Response selection difficulty during learning did not interact with any of the other factors, all *F* < 1.1, *p* > 0.30.

**Table 1:** Mean reaction time (RT) and accuracy for compatible (C) and incompatible (I) test trials with and without response cues (RCs), along with mean compatibility effects (I-C).

	With RC			Without RC		
	C	I	I-C	C	I	I-C
**Response times (ms)**
Experiment 1: spatial	632	635	2.8	673	684	10.8***
Fully guided	645	649	4.5*	694	707	12.6**
Partly guided	619	620	1.2	653	662	9.0*
Experiment 2:
Non-spatial	740	762	22.2***	682	704	21.5***
Experiment 3:
Spatial, long delay	885	898	13.2**	821	835	13.6*
**Accuracy (%)**
Experiment 1: spatial	98.4	98.3	–0.1	91.3	88.3	–3.3***
Fully guided	98.7	98.2	–0.5	93.1	89.7	–3.4***
Partly guided	98.2	98.4	–0.2	89.6	86.9	–2.7**
Experiment 2:
Non-spatial	94.9	89.9	–5.0***	89.7	86.3	–3.4***
Experiment 3:
Spatial, long delay	98.7	97.0	–1.7**	92.4	88.9	–3.5***

Asterisks indicate significant compatibility effects as revealed by one-sided *t*-tests, *p < 0.05, **p < 0.01, ***p < 0.001. Note that the significant compatibility effect on RT in the fully guided spatial group in Experiment 1 is most likely a spurious effect. There was no three-way interaction of response selection difficulty at test, compatibility and response selection difficulty during learning and averaged across both groups, the effect was non-significant (*M* = 2.6 ms, SD = 13.2). Furthermore, the effect was considerably smaller than all others. Finally, when splitting up the experiment in two halves (see **Table 2**) the RT compatibility effect was not significant in either half.

Unsurprisingly, accuracy was higher in trials with RCs (98.4%) compared to trials without RCs (89.8%), *F*(1,48) = 117.1, MSE = 31.53, *p* < 0.001, $\eta_{p}^{2}$ = 0.71. Responses on compatible trials (94.9%) were slightly more accurate than responses on incompatible trials (93.3%), *F*(1,48) = 26.4, MSE = 4.71, *p* < 0.001, $\eta_{p}^{2}$ = 0.36. However, as for RT there was also a significant interaction^2^ of compatibility and response selection difficulty during test, *F*(1,48) = 26.0, MSE = 4.07, *p* < 0.001, $\eta_{p}^{2}$ = 0.35, and paired *t*-tests revealed a reliable compatibility effect on accuracy for the test trials without RCs only (see **Table 1** and **Figure 2**). Again, response selection difficulty during learning did not further qualify any effect involving compatibility, all (*F* < 1.3, *p* > 0.27).

#### Behavioral expression of action effects: stability

To explore whether experiencing multiple learning episodes influences the rapid learning or expression of action effect associations, we split the experiments in two halves and reran the above analyses including experiment-half as an additional within-subject factor. Accordingly the focus of the analysis is on interactions involving compatibility and experiment-half. For RT, a significant decrease in compatibility effects in the second half of the experiment was observed, *F*(1,48) = 6.71, MSE = 354.95, *p* < 0.05, $\eta_{p}^{2}$ = 0.12. While for the first half, significant compatibility effects were observed in trials without RCs, they virtually disappeared in the second half of the experiment (see **Table 2**). Similarly, for accuracy, experiment half was involved in a two-way interaction with compatibility, *F*(1,48) = 9.3, MSE = 5.93, *p* < 0.01, $\eta_{p}^{2}$ = 0.16, and a three-way interaction with compatibility and response selection difficulty at test, *F*(1,48) = 7.6, MSE = 4.30, *p* < 0.01, $\eta_{p}^{2}$ = 0.14 (see **Table 2**).

**Table 2:** Mean compatibility effects for RT and accuracy for test trials with and without RC are given for the first and second half of the experiment.

	Block 1–10		Block 11–20	
	With RC	Without RC	With RC	Without RC
**Compatibility effects (I-C) in RT (ms)**
Experiment 1:
Fully guided spatial	6.5	18.6**	2.6	6.6
Partly guided spatial	4.8	17.1**	–2.3	0.9
Experiment 2:
Non-spatial	27.7**	16.6*	16.6**	26.5***
Experiment 3:
Spatial, long delay	17.2*	25.0**	9.2	2.2
**Compatibility effects (I-C) in accuracy (%)**
Experiment 1:
Fully guided spatial	–0.8*	–4.4***	–0.1	–2.3**
Partly guided spatial	0.2	–4.3***	0.2	–1.1
Experiment 2:
Non-spatial	–7.0***	–4.5***	–3.0**	–2.6**
Experiment 3:
Spatial, long delay	–1.6*	–4.5***	–1.8**	–2.6***

Asterisks indicate significant compatibility effects as revealed by one-sided *t* -tests, *p < 0.05, **p < 0.01, ***p < 0.001.

### DISCUSSION

The results of Experiment 1 lend further support to the notion that action effect associations are acquired not only when responses are freely chosen, but also when responding according to an S–R rule (Pfister et al., 2011; Wolfensteller and Ruge, 2011). But more importantly, the present results add two novel insights. First, the strength of the learnt action effect association is not affected by response selection difficulty during learning. Even in the fully guided group, when spatial RCs automatically activate the corresponding response in all learning trials action effects are rapidly acquired. Second, action effect associations seem to affect behavior only when there is some demand for active response selection. This was the case when the newly established mapping linking former effects to responses had to be retrieved without the help of additional RCs. In turn, when response selection demands were very low in trials with spatial RCs, the previously learnt action effect associations did not systematically influence overt behavior.

A noteworthy aspect of the present data is that in the second half of the experiment, action effect expression was significantly decreased. Though we cannot conclude whether action effects were no longer learnt or were more efficiently suppressed during the test phase it seems reasonable to assume that any strategic change occurred in the fully and partly guided group alike.

## EXPERIMENT 2

Experiment 2 aimed to further substantiate the notion that response selection difficulty moderates the behavioral expression of learnt action effect associations. We hypothesized that non-spatial RCs which pose higher demands on response selection than spatial RCs would allow for a behavioral expression of the previously learnt action effect associations. Thus, we expected significant compatibility effects in test trials with non-spatial RCs, and, as replication of Experiment 1, in test trials without RCs.

### MATERIAL AND METHODS

#### Participants

A new sample of 25 young adults participated in the experiment (six male, mean age 23.6 years) and received monetary compensation (5 20AC) or course credit. However, due to technical problems one dataset was lost, and one had to be excluded due to excessive error rates (>65%) resulting in a final sample size of 23 ^3^.

#### Stimuli and procedure

Stimuli and procedure were comparable to Experiment 1. The only difference was that participants received non-spatial RCs (i.e., central presentation of the key letter with which to respond D, F, K, L), again only for the first three repetitions of each stimulus (*partly guided non-spatial*).

### RESULTS

#### Learning of action effects

First we determined whether stimulus repetition 4 was associated with a dip in accuracy compared to stimulus repetition 3 as in the partly guided group of Experiment 1. This was the case, accuracy temporarily dropped from 92.1 to 85.3%, *t*(1,22) = 3.50, *p* < 0.01, thus indicating that participants had used the RCs. In order to confirm that response selection was more difficult based on non-spatial compared to spatial RCs we then directly compared RT and accuracy in Experiment 2 with the partly guided group of Experiment 1. It turned out that participants responded significantly slower (127 ms) and less accurate (5.9%) with non-spatial RCs than with spatial RCs for the first three stimulus repetitions, *t*s > 4.32, *p* < 0.01(Bonferroni-corrected). In contrast, there was no difference for the later stimulus repetition levels, when no additional RCs were provided, all *t*s < 1.2, *p* > 0.05 (Bonferroni-corrected). This clearly indicates that although response selection based on non-spatial RCs was more difficult, spatial, and non-spatial RCs were of comparable instructional value to the participants.

.

#### Behavioral expression of action effects

**FIGURE 3:**
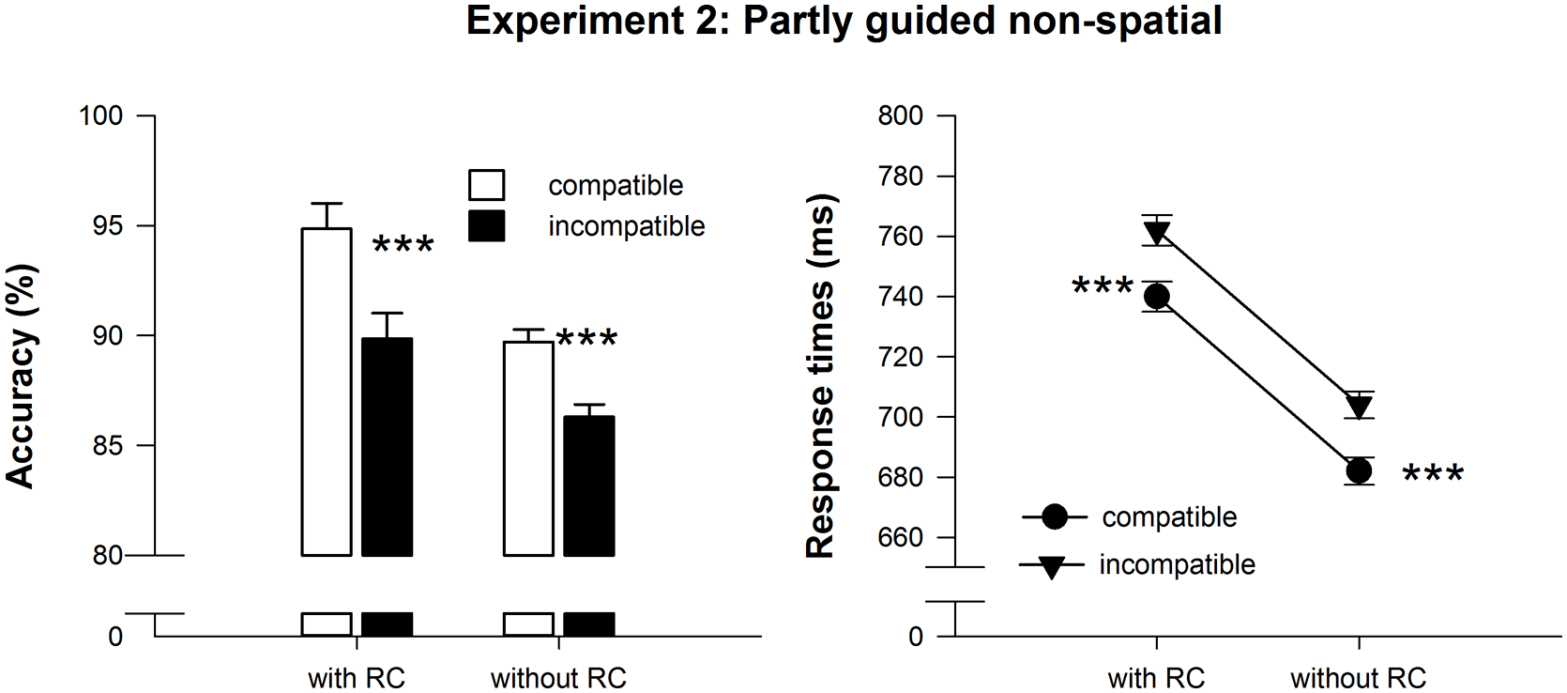
**Mean reaction time and accuracy data of the test phase in Experiment 3.** Error bars denote SE_PD_, (Pfister and Janczyk, 2013). Asterisks denote significantly longer or less accurate responses for incompatible compared to compatible effect-response mappings, *p < .05, **p < 0.01, ***p < 0.001, all one-sided paired *t*-tests.

Repeated measures ANOVAs on RT and accuracy revealed main effects of compatibility [RT: *F*(1,22) = 32.48, MSE = 338.03, *p* < 0.001, $\eta_{p}^{2}$ = 0.60, accuracy: *F*(1,22) = 35.38, MSE = 403.36, *p* < 0.001, $\eta_{p}^{2}$ = 0.62] and response selection difficulty at test, [RT: *F*(1,22) = 17.41, MSE = 4400.81, *p* < 0.001, $\eta_{p}^{2}$ = 0.44 accuracy: *F*(1,22) = 15.95, MSE = 27.29, *p* < 0.01, $\eta_{p}^{2}$ = 0.42], but no interaction of the two factors [RT: *F*(1,22) = 0.01, MSE = 2.30, *p* > 0.90, accuracy: *F*(1,22) = 1.91, MSE = 7.64, *p* > 0.17]. As predicted, responses on compatible trials were delivered faster (711 ms) and more accurately (92.3%) than responses on incompatible trials (733 ms, 88%). Importantly, in line with our hypothesis, the compatibility effect was present in test trials with and without RCs in RT and accuracy, *t*(22) > 4.3, *p* < 0.001 (see also **Table 1** and **Figure 3**). As in Experiment 1, responses were more accurate (92.4%) in test trials with RCs compared to trials without RCs (88%). But unlike in Experiment 1, responses were slower in test trials with RCs (751 ms) than in trials without RCs (693 ms). This demonstrates that response selection based on non-spatial RCs was indeed more difficult.

#### Behavioral expression of action effects: stability

To explore whether response selection difficulty might modulate the impact of experiencing multiple learning episodes on rapid learning or expression of action effect associations, we split the experiments in two halves and reran the above analyses including experiment-half as an additional within-subject factor. Compatibility effects in accuracy generally decreased from the first (*M* = 4.5%, SD = 5.4%) to the second half of the experiment (*M* = 3.0%, SD = 5.2%) as reflected by a compatibility × experiment half interaction, *F*(1,22) = 8.27, MSE = 13.13, *p* < 0.01, $\eta_{p}^{2}$ = 0.27. For RT, compatibility did not interact with experiment half, *F*(1,22) = 0.005, MSE = 773.59, *p* > 0.90. Thus, unlike in Experiment 1, compatibility effects remained virtually unchanged across the experiment (cf. **Table 2**).

To substantiate these differences between Experiments 1 and 2, a repeated measures MANOVA on compatibility effects in RT and accuracy was performed, which revealed a three-way interaction of experiment (spatial vs. non-spatial RCs), response selection difficulty at test (test trials with and without RCs), and experiment half, *F*(2,45) = 3.9, *p* < 0.05, $\eta_{p}^{2}$ = 0.15. Compatibility effects in trials with RCs were always larger with non-spatial RCs (Experiment 2), *t* > 2.37, *p* < 0.05. The compatibility effect in trials without RCs did not differ between participants who had received non-spatial (Experiment 2) and spatial RCs (Experiment 1) in the first half of the experiment (*t* < 0.20, *p* > 0.90). In contrast, in the second half of the experiment, the RT compatibility effect was significantly larger in participants who had received non-spatial RCs (Experiment 2), *t* = 2.87, *p* < 0.01.

#### Behavioral expression of action effects: first block of learning

As a subsidiary analysis, we also checked for compatibility effects in the very first test block, where learning could not possibly have been influenced by having experienced a test phase yet. To increase statistical power, RT and accuracy data were collapsed across groups and experiments, because otherwise the individual cell means contained no more than 12 trials each. Paired *t*-tests revealed significant compatibility effects on both, RT [*M* = 24.6 ms, SD = 122.5 ms, *t*(72) = 1.72, *p*_one-sided_ < 0.05], and accuracy [*M* = 5.3%, SD = 18.1%, *t*(72) = 2.5, *p*_one-sided_ < 0.01].

#### Behavioral expression of action effects: influence of RT level

To rule out that differences between the partly guided groups in Experiments 1 and 2 could be explained by general differences in RT-levels (see also **Table 1**), we performed additional quintile analyses. To this end, individual RTs were rank-ordered separately for compatible and incompatible trials with and without RCs. In light of the above reported reduction of the compatibility effects in the second half of the experiment, we restricted this analysis to the first half of the experiment.

**FIGURE 4:**
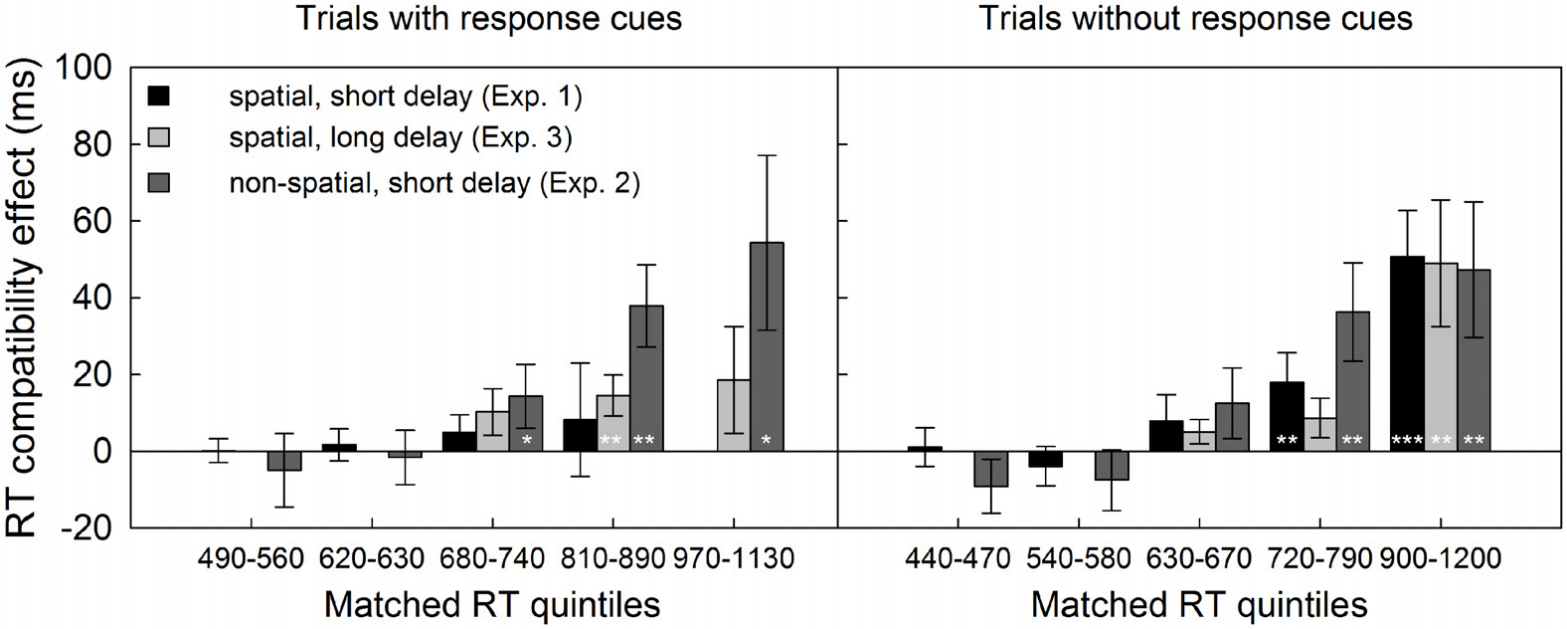
**Reaction time quintile analysis across experiments.** Individual RTs were rank-ordered and binned into quintiles, separately for compatible and incompatible test trials with and without response cues. The bins extracted for Experiments 1 and 3 which spanned a more limited range in RT were sorted and averaged into the five bins extracted for Experiment 2. Consequently, empty cells resulted for Experiment 1 at the higher end of the RT distribution (no RTs between 970 and 1130 ms in trials with response cues) and for Experiment 3 at the lower tail of the distribution (no RTs below 630 ms in test trials with and without response cues). Bars depict compatibility effects in ms, error bars represent the standard error of the mean SE_M_, *p < 0.05, **p < 0.01, ***p < 0.001, all one-sided paired t-tests.

Interestingly, a significant three-way interaction of response selection difficulty at test, compatibility and quintile was revealed for Experiment 1, *F*(1.4,34.2) = 3.93, MSE = 1518.84, *p* < 0.05, $\eta_{p}^{2}$ = 0.14, but not for Experiment 2, *F*(1.7,38.3) = 0.04, MSE = 2061.71, *p* > 0.90. More specifically, in Experiment 1, the compatibility effect increased with RT in trials without RCs, *F*(1.7,41.2) = 11.12, MSE = 1240.33, *p* < 0.001, $\eta_{p}^{2}$ = 0.32 but not in trials with spatial RCs, *F*(1.2,29.3) = 0.26, MSE = 1980.04, *p* > 0.65 (see **Figure 4**). In contrast, in Experiment 2, the compatibility effect increased with RT similarly in trials with and without non-spatial RCs, *F*(1.6,35.2) = 10.7, MSE = 3477.21, *p* < 0.01, $\eta_{p}^{2}$ = 0.34. Moreover, the mean compatibility effect in the slowest test trials with spatial RCs was not reliable [Experiment 1, 8 ms, *t*(24) = 0.56, *p* > 0.25], and about four times smaller than the one in RT-matched second-slowest quintile of test trials with non-spatial RCs [Experiment 2, 38 ms, *t*(46) = 1.60, *p*_one-sided_ = 0.055, see also **Figure 4**]. Together, this suggests that the effect of response selection difficulty on compatibility cannot be explained by differences in RT level alone.

### DISCUSSION

Experiment 2 corroborates and extends the findings of Experiment 1. In line with our hypotheses, learnt action effect associations affected behavior in test trials with and without additional RCs. In Experiment 1, the RCs allowed for automatic response activation as in most stimulus-based conditions in previous studies that did not report action effect learning. In line with these studies, no compatibility effect was observed in trials with spatial RCs. In contrast, in Experiment 2, response selection was more difficult because the correct response to the non-spatial RCs had to be actively retrieved. As a consequence, a significant compatibility effect emerged. We suggest that the need for active response retrieval constitutes an action mode in which previously learnt action effect associations can affect behavior. Experiment 2 also revealed that when responses were instructed via cues that did not allow for direct response activation, the behavioral expression of action effects was more stable across the experiment than in Experiment 1. However, before discussing the putative mechanisms underlying this difference we have to consider an alternative explanation for the difference in compatibility effects.

Response times in trials with RCs were substantially longer in Experiment 2 than in Experiment 1 (see **Table 1**). Importantly though the effect of response selection difficulty cannot be attributed to differences in RT level alone for two reasons. Even at the slowest RT level, no substantial compatibility effect was observed in trials with spatial RCs. In contrast, without RCs, or with non-spatial RCs, pronounced and larger compatibility effects emerged. Nevertheless, time seems to be a contributing factor as compatibility effects became increasingly stronger with longer RT which is in line with studies using the response-effect compatibility paradigm (Kunde, 2001; Paelecke and Kunde, 2007; Pfister et al., 2014a). In order to test more directly whether time alone can account for the pattern of results observed with higher response selection difficulty we conducted a third experiment.

## EXPERIMENT 3

In Experiment 3 we wanted to elaborate whether giving the effect more time to prime their associated action would yield the same pattern of results as increasing response selection difficulty.

### MATERIALS AND METHODS

#### Participants

A new sample of 25 young adults participated in the experiment (11 male, mean age 22.7 years) and received monetary compensation (5 20AC) or course credit.

#### Stimuli and procedure

Stimuli and procedure were identical to the partly guided spatial group in Experiment 1. The only difference pertained to the test phase where the delay between the onset of the former effect sound and the spatial RCs was increased to 450 ms. Again, response time was always calculated in reference the onset of the visual or sound stimulus.

### RESULTS

#### Learning of action effects

As in Experiments 1 and 2, accuracy temporarily dropped from 98.7 to 87.8% [*t*(24) = 5.64, *p* < 0.001] from stimulus repetition 3–4, thus indicating that participants used the RCs. When comparing Experiment 3 with the partly guided spatial group in Experiment 1, a main effect of experiment on RT indicated a general slowing of 36.2 ms in Experiment 3 [*F*(1,48) = 5.22, MSE = 25136.69, *p* < 0.05, $\eta_{p}^{2}$ = 0.10], but there was no interaction of stimulus repetition and experiment (*F*s < 1, *p* > 0.85).

#### Behavioral expression of action effects

**FIGURE 5:**
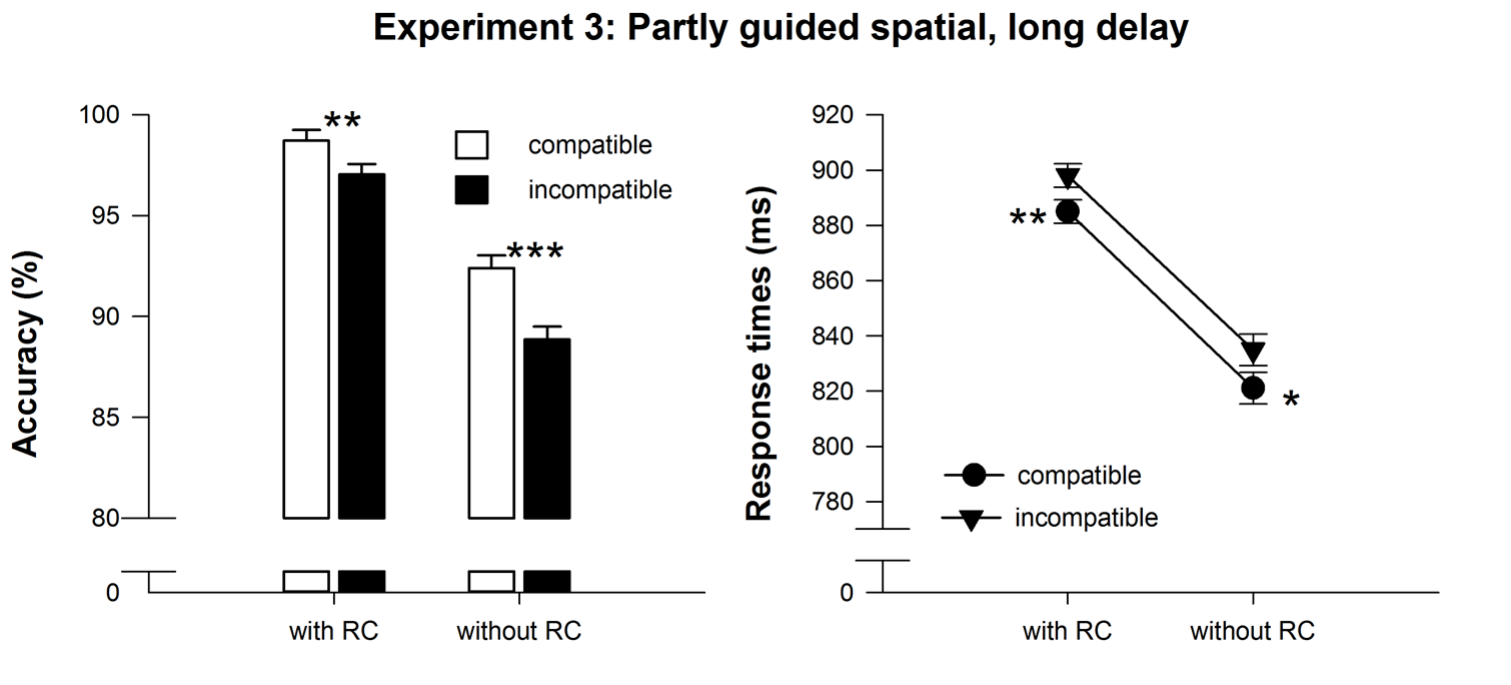
**Mean reaction time and accuracy data of the test phase in Experiment 3.** Error bars denote SEPD, (Pfister and Janczyk, 2013). Asterisks denote significantly longer or less accurate responses for incompatible compared to compatible effect-response mappings, *p < .05, **p < 0.01, ***p < 0.001, all one-sided paired t-tests.

Repeated measures ANOVAs on RT revealed main effects of compatibility, *F*(1,24) = 9.87, MSE = 454.85, *p* < 0.01, $\eta_{p}^{2}$ = 0.29, and response selection difficulty at test, *F*(1,24) = 24.7, MSE = 4109.98, *p* < 0.001, $\eta_{p}^{2}$ = 0.51, but no interaction, *F*(1,24) = 0.05, MSE = 177.36, *p* > 0.90. Participants’ responses with delayed RCs were significantly slower (892 ms) than in trials without RCs (828 ms). Furthermore, responses in compatible trials were significantly faster (853 ms) than in incompatible trials (867 ms). The compatibility effect was present in trials with and without RCs (*t*_24_ > 2.3, *p*_one-sided_ < 0.03, see **Table 1** and **Figure 5**). Unsurprisingly, responses were more accurate with RCs (97.9%) than without RCs (90.6%), *F*(1,24) = 49.1, MSE = 26.78, *p* < 0.001, $\eta_{p}^{2}$ = 0.67. Moreover, accuracy was higher on compatible (95.6%) compared to incompatible trials (93.0%), *F*(1,24) = 35.9, MSE = 4.75, *p* < 0.001, $\eta_{p}^{2}$ = 0.60. Again, the compatibility effect was present in trials with and without RCs (*t*_24_ > 3.2, *p*_one-sided_ < 0.01, see **Table 1** and **Figure 5**), but it was more pronounced in trials without RCs, *F*(1,24) = 5.8, MSE = 3.66, *p* < 0.05, $\eta_{p}^{2}$ = 0.20. While the compatibility effect on accuracy remained unchanged across the experiment, the RT compatibility effects virtually vanished in the second half of the experiment, *F*(1,24) = 1.19, MSE = 687.9, *p* > 0.25.

#### Behavioral expression of action effects: influence of RT level

A quintile analysis replicated the increase of the compatibility effect across RT bins for trials without RCs observed in Experiments 1 and 2, *F*(1.4,33.6) = 7.27, MSE = 3209.88, *p* < .01, $\eta_{p}^{2}$ = 0.23. In contrast, in trials with delayed RCs, the compatibility effect did not increase with RT level, *F*(1.6,38.9) = 0.30, MSE = 2020.19, *p* > 0.65. Importantly, the compatibility effect was significantly larger in trials without RCs than in trials with RCs at the two slowest RT-levels [65 vs. 22 ms, and 38 vs. 14 ms, (24) > 1.72, *p*_one-sided_ < 0.05]. To account for general differences in RT level between experiments, we compared the compatibility effects in matched bins. At the slowest matched bin, compatibility effects with spatial RCs were of the same size in Experiments 1 and 3, irrespective of the delay (8 ms with 150 ms delay, 15 ms with 450 ms delay, *t* = 0.43, *p*_one-sided_ > 0.30). Of note, both of these effects were non-significant themselves (see also **Figure 4**). In contrast, the compatibility effects in the two slowest bins with non-spatial RCs (Experiment 2, 54 and 38 ms) were two to three times larger than with delayed spatial RCs (Experiment 3, 15 ms, *t* = 1.55, *p*_one-sided_ = 0.065, and *t* = 1.98, *p*_one-sided_ < 0.05).

### DISCUSSION

Experiment 3 aimed to directly test whether the influence of response selection on the expression of action effects as observed in Experiments 1 and 2 can be explained solely by differences in RT level. To this end, the response-guiding spatial cue was delayed by 450 ms after stimulus onset, compared to a short delay of only 150 ms in Experiments 1 and 2. The results are straightforward. In trials with delayed spatial RCs, behavior was influenced by action effect compatibility. But although the compatibility effects in Experiments 2 and 3 were of comparable overall size, a closer inspection of RT distribution suggests that they are brought about by markedly different mechanisms.

With spatial RCs, the compatibility effect was not further influenced by the individual RT level. If the delay was short as in Experiment 1, there was no compatibility effect. If the delay was longer as in Experiment 3, a moderate compatibility effect emerged. In contrast, if response selection demands were high, as in trials with non-spatial RCs in Experiment 2, and in trials without RCs in all three experiments, a compatibility effect emerged and continually increases in trials with longer RTs. Importantly, the size of the latter compatibility effect induced by response selection difficulty substantially surmounts the one induced by prolonging the temporal delay between effect prime and RC.

## GENERAL DISCUSSION

The aim of the present study was to shed light on the influence of response selection difficulty as one aspect of rule complexity on the rapid learning and expression of action effects in situations where action selection is (at least partly) based upon external stimuli as is quite commonly the case in everyday life. We conducted three experiments using the rapid action effect learning paradigm, a variant of the classic two-phase action effect induction paradigm (Greenwald, 1970; Hommel, 1996; Elsner and Hommel, 2001). In a nutshell, participants perform several unique blocks of short action effect learning and test phases. In both phases, participants are instructed about the correct S–R rule by RCs provided shortly after the stimulus. In the first experiment, we manipulated response selection difficulty during the learning phase by providing a different number of trials guided by spatial RCs in two groups of participants. During the test phase, response selection difficulty differed similarly between trials with and without RCs in both groups. In the second experiment we used non-spatial RCs to prevent direct response activation and evoke more difficult response selection in both acquisition and test phase. In the third experiment we introduced a longer delay between former action effects and RCs in the test trials in order to dissociate the influence of response selection difficulty from an influence of elapsed time.

Generally, the present experiments clearly show that learning of action effects is a very rapid process, which replicates our own previous findings (Wolfensteller and Ruge, 2011; Ruge et al., 2012) and is also in line with recent empirical findings on very short-term within-trial action effect binding (Dutzi and Hommel, 2009; Herwig and Waszak, 2012; Janczyk et al., 2012). More specifically, the present results clearly show that higher response selection difficulty does not directly boost action effect *learning*. In contrast, it does affect the *behavioral expression* of action effects. When response selection was difficult, previously learnt action effect associations influenced behavior such that responding in an effect-compatible manner was faster than responding in an effect-incompatible manner. Interestingly, with low response selection difficulty, a smaller behavioral influence of action effects could be induced by delaying the information that made response selection easy.

### RESPONSE SELECTION DIFFICULTY INFLUENCES ACTION EFFECT EXPRESSION, BUT NOT LEARNING

Three aspects of the present results dismiss a direct influence of response selection difficulty on action effect learning. First, in Experiment 1 action effects were learned even when responding was fully guided by spatial RCs and hence there was no need to ever learn or retrieve S–R rules during learning. Second, the compatibility effects indicating action effect learning were of comparable size in the group who always received RCs and the group who received them only for the first couple of trials as instruction. Third, the partly guided spatial group of Experiments 1 and 3, and the partly guided non-spatial group of Experiment 2 displayed compatibility effects of similar size in test trials without RCs in the first half of the experiment. Thereby the present results further substantiate the notion that action effect learning does not depend on an intentional mode of action control at least not in the sense of being purely endogenously driven as has previously been suggested (Herwig et al., 2007; Herwig and Waszak, 2009).

Our findings also contrast with the assumption that the behavioral expression of action effects requires an intentional free-choice action mode (Pfister et al., 2011). If that were true, none of the experiments should have revealed a compatibility effect of any sort. Based on our findings, we propose instead that response selection difficulty contributes to the behavioral expression of learnt R-E associations. When response selection was difficult – either because no additional RCs were given, or because the RCs did not automatically activate a response due to dimensional overlap or overtraining – the learnt action effect associations influenced behavior in the predicted manner. This fits with recent results by Ziessler et al. (2012) who studied the behavioral impact of response-effect associations evoked by merely anticipating the effect. In an easy version of their task (Experiment 1), participants learnt associations between two freely chosen button presses and two effects. Then they had to prepare responses to one of two stimuli, but execution depended on a Go-signal which was semantically related to the previously acquired action effects, and compatible or incompatible with respect to the currently required response. Interestingly, roughly one third of the participants showed no compatibility effect. Importantly, these participants produced a substantial amount of false alarms on No-Go trials, and responded substantially faster on Go-trials than the other participants. In fact, without time for response preparation they responded just as fast as the other participants when those had the most time for response preparation. Thus it seems reasonable to assume that the former did not base response selection and execution on the combination of stimulus and Go-signal as instructed, but rather on the stimulus alone and thereby effectively reduced the complexity of the choice. When making the rule more complex, by using four stimuli, four responses, six go/no-go stimuli and four effects (Experiment 3), the expected compatibility effects were reliably observed in all participants.

Our findings also fit with research on the differential outcome effect in humans. In this domain it has been shown that the beneficial effect of receiving choice specific outcomes on learning and memory depends on the complexity of the choice problem (Estevez et al., 2001; Legge and Spetch, 2009; Plaza et al., 2011). In fact, while a differential outcome effect could be obtained in young children (Estevez et al., 2001; Legge and Spetch, 2009; Martinez et al., 2009) and adults with mental problems (e.g., Estevez et al., 2003; Plaza et al., 2012), at first, it appeared to be absent in older children and healthy adults (Estevez et al., 2001; Legge and Spetch, 2009; Sturz et al., 2012). Yet, when choice problems are sufficiently hard, either because the stimuli are hard to discriminate or because there are more than just two choice options, adults benefit from the differential outcomes, i.e., specific response-effects (Estevez et al., 2001; Miller et al., 2002; Mok and Overmier, 2007; Legge and Spetch, 2009; Mok et al., 2009; Plaza et al., 2011). In light of the present findings it seems reasonable to assume that differential outcomes are always learnt, and their beneficial effect on overt behavior is only masked in choices that are too simple.

Can response selection difficulty account for the fact that action effects are reliably learned and expressed in free-choice actions? We argue that it can. In most studies, the free-choice instruction is to select a response anew on each trial, e.g., as if ‘tossing a coin,’ but to produce each response about equally often (e.g., Elsner and Hommel, 2001). Participants are able to adhere to the required constraints and produce a near-equal distribution of left and right button presses. But such freely chosen actions have been described as ‘underdetermined’ because of the absence of external cues, ‘operant upon internal cues’ such as memory traces and past actions and the ‘result of a complex integration of different types of information’ (see Schuur and Haggard, 2011). These terms at least imply that free-choice response selection is less easy than pressing a left button in response to a left-pointing arrow. The documented difficulties humans encounter when producing or recognizing random sequences (see e.g., Wagenaar, 1972; Nickerson, 2002) further suggest that the free-choice instruction constitutes a rather complicated rule. Thus, response selection under free-choice instructions is more difficult and there is clearly no possibility for direct response activation as in the simple S–R rules typically used to investigate stimulus-based actions.

### AN INDIRECT INFLUENCE OF RESPONSE SELECTION DIFFICULTY ON RAPID ACTION EFFECT LEARNING?

Experiment 1 indicates that at least for easy S–R associations such as spatial RCs, the impact of R-E associations on overt behavior decays over the course of the experiment. The most straightforward explanation for this finding is the sheer amount of different R-E associations that have been learnt (and revised) by this time. After half of the experiment, participants had learnt 10 effects for each of four responses adding up to a total of 40 completely task-irrelevant action effect associations. This stands in stark contrast to the two action effect associations typically learnt in other versions of the induction paradigm (Hommel, 1996; Elsner and Hommel, 2001; Herwig et al., 2007; Herwig and Waszak, 2009; Verschoor et al., 2011; Ziessler et al., 2012). It seems reasonable to assume that the salience of stimuli serving as action effects diminishes if the same responses are paired with more and more different effects thus hampering or preventing learning of irrelevant novel action effect associations. However, in Experiment 2 when response selection difficulty was high during both learning and test phase, learnt action effects affected behavior to a virtually unchanged degree across the experiment. This strongly suggests that the mere number of different action effect associations does not by itself prevent action effect learning.

Alternatively, different degrees of rule difficulty might induce different strategies. The decreasing impact of action effect on behavior across the course of the experiment might reflect a trade-off between exploring novel action-related environmental contingencies and ignoring them in the light of mounting evidence of their absolute irrelevance, and their even partially detrimental effects on behavior (in the case of incompatible mappings between former action effects and novel actions in the test phase). If the instruction of novel rules is fairly direct as with spatial RCs, an effective strategy would be to rely more and more on the stimulus-side of the rule and to actively ignore or suppress the subsequent action effect. This relates to compression of event files according to intentional weighting (see e.g., Herwig and Waszak, 2012). If however, response selection is more difficult, then using the anticipated action effect as another retrieval cue in the sense of a differential outcome might be more efficient (cf. Estevez et al., 2001; Legge and Spetch, 2009; Plaza et al., 2011). In that sense, response selection difficulty might exhibit an indirect effect on action effect learning, by biasing strategies toward using or suppressing them. However, this strategy seems to become relevant only after encountering what might putatively be an overwhelming number of novel (and irrelevant) action effect associations in a very short period of time.

### IS RESPONSE SELECTION DIFFICULTY MORE THAN JUST A LITTLE MORE TIME?

By definition, increasing response selection difficulty, either by increasing the number of choice options or by using arbitrary rather than spatially compatible stimuli, increases response time. Priming in general, and more specifically, effect-based priming, has been shown to increase with response time (Hommel, 1996; Paelecke and Kunde, 2007). This leads to the question whether and in what way the effect of response selection difficulty differs from giving effects more time to prime the action.

A closer inspection of the compatibility effects in different parts of the response time distributions of all three experiments suggests an influence of response selection difficulty different from and above the influence of just more time itself. Compatibility effects were indeed more pronounced for slower responses. Importantly though, this was restricted to conditions which posed some demands on response selection, such as trials without RCs in all three experiments, or trials with non-spatial RCs in Experiment 2. Increasing the temporal delay between stimulus and spatial RC – thereby effectively increasing response times and time for effect-priming above the level induced by non-spatial RCs – yielded a different pattern in Experiment 3. The induced compatibility effects were substantially smaller than in RT-matched trials posing higher demands on response selection, and they did not increase with RT level.

What does that imply for the contributions of time and response selection difficulty to the behavioral expression action effects? Let’s consider the effect of time first. Spatial RCs prevent action effects from affecting behavior when they are presented at a short, but not at a long delay after the relevant stimulus. Thus, the effect of action effect compatibility is under-additive with the effect of the delay between stimulus and response-cue onset. In terms of the locus-of-slack logic this suggests overlap at parallel stages of processing (Hommel, 1998; Lien and Proctor, 2002; Janczyk et al., 2014). In the present study, the effects were rather complex natural sounds which could not be discriminated by simply attending to one pop-out feature such as pitch, which is typically used (Elsner and Hommel, 2001; Herwig et al., 2007). It is thus reasonable to assume that late perceptual processing (putatively stimulus classification, Johnston and McCann, 2006) of the former action effect was relatively prolonged leading to a postponement of the successive response activation. As a consequence, the more easy-to-discriminate spatial RC could beat the former effect sound to the response selection stage. Because the response activation stage for the action effect was postponed, there was no crosstalk at this stage, and thus no compatibility effect ensued in Experiment 1. When response activation for the spatial RC was shifted in time such that the perceptual processing of the former action effect was more advanced, crosstalk at the response activation stage could occur which explains the action effect compatibility effect in Experiment 3.

When response selection was more difficult, action effect compatibility influenced responses at the slower tail of the response distribution more severely than when response selection was easy. Based on the rationale just outlined this cannot be explained by a temporal shift of response activation for the relevant stimulus dimension. It seems more in line with research on the differential outcome effect in humans suggesting that specific action effects are actively used when response selection is difficult (Estevez et al., 2001; Legge and Spetch, 2009; Plaza et al., 2011). This would result in two equally strong response tendencies at the response activation stage, i.e., the previous effect-response and the currently relevant S–R association. This explains the relatively enhanced compatibility effect. The fact that the RT compatibility effect was not evident at the fast tail of the response distribution and increased with RT resembles findings in the response-effect compatibility task (Kunde, 2001; Paelecke and Kunde, 2007; Pfister et al., 2014a), and, though not as commonly probed, findings on action effect induction (see e.g., Hommel, 1996), as well as more generally, Stroop tasks and certain variants of the Simon Task (Pratte et al., 2010). In the present study it most likely reflects the fact that effect discrimination was prolonged because of the complexity of the sounds. Sounds lasted for 500 ms and the earliest bin yielding significant compatibility effects comprised RTs between 680 and 740 ms after stimulus onset. The faster two RT bins might then reflect either less thorough or more focused stimulus processing indicative of a strategic bias, possibly not unlike the one resulting in a decreased impact of action effects after experiencing multiple rapid learning and relearning episodes.

### WHAT OTHER MECHANISMS MIGHT CONTRIBUTE TO DISCREPANCIES IN ACTION EFFECT EXPRESSION?

Here we have considered the influence of response selection difficulty, one aspect of rule complexity, to the behavioral expression of rapidly learnt action effect associations. There are, however, several other mechanisms that might principally contribute to the striking discrepancies in studies investigating action effect learning when reacting to a stimulus according to a rule.

First, in our rapid action effect learning paradigm, participants had to repetitively switch between learning and test which might have rendered action effects more relevant thus boosting learning (as suggested by Herwig and Waszak, 2012). Since our initial instructions stated that participants would have to respond to the sounds at some point, we cannot completely rule out that effects became more relevant than in other studies, but Herwig and Waszak (2009) have already shown that behavioral relevance *per se* does not boost action effect learning. However, we can rule out that intermixing of learning and test-phase is an explanation for the discrepant findings. In the present study, learnt action effect associations affected behavior even when no test phase was ever experienced before. Furthermore, while responses get increasingly faster when a novel S–R is practiced, a relative slowing of this speed-up seems to be related to action effect learning (Ruge et al., 2012). Interestingly, this relative slowing in turn was also present in the absence of intermixed test-phases (Ruge and Wolfensteller, 2013).

Another aspect related to the design of the rapid action effect learning paradigm is the brevity of our learning and test phases. Associations of the former effects with the newly instructed responses naturally get increasingly stronger which over time should counteract the compatibility effect. Here, we used very short learning test phases (nine repetitions per stimulus) but Ziessler et al. (2012) reported at least a numerical decrease in the compatibility effect across the course of their test phase. It seems reasonable to assume that a system able to quickly pick up completely arbitrary S-R-E associations should be able to drop them just as quickly. Note that we did not present the previous action effects after the response in the test phase. Thus, by the end of the test phase participants had as many experiences of an action not being followed by an effect as they had previous experiences of that action being followed by a specific effect – thus the length of our test phase might have been just short enough to still detect the impact of the rapidly learnt action effects. Alternatively, our learning phase might have been just short enough for the action effect associations to still be able to affect behavior. This would be in line with the intentional weighting and compression account proposed by Herwig and Waszak (2012). They suggested that in the course of learning an originally very broad event-file containing the action and the preceding and following environmental specifications gets compressed by such that it contains only the relevant associations. In stimulus-based action modes this means putting weight on the S–R associations and ignoring the response-effect association. The opposite holds for the free-choice action mode. As a result, with increasing practice action effect associations should cease to affect behavior. In combination those mechanisms might account for the previous finding that the same simple two-choice test phase procedure revealed an impact of action effects on behavior after a free-choice learning phase (Elsner and Hommel, 2001; Herwig et al., 2007) but not after a forced-choice learning phase (Herwig et al., 2007) which cannot readily be explained by response selection difficulty alone.

Finally, in our paradigm compatibility is manipulated within participants, because each test block contains effect-compatible and effect-incompatible responses. In other studies response-effect compatibility has typically been manipulated between subjects (Elsner and Hommel, 2001; Herwig et al., 2007; Herwig and Waszak, 2009). Under these circumstances, participants in the incompatible group might have put in more effort to overcome the response bias induced by the previous response-effect association which could in principle abolish the compatibility effect. Due to our experimental design, such an overall strategy would not have been an efficient strategy as performance would actually benefit from the response bias in compatible trials.

Note however, that the latter two explanations apply to action effect associations acquired in an intention-based action mode as well, yet these are apparently mostly and maybe surprisingly unaffected by these factors. As a matter of fact, in these studies, the compatibility of the (former) effect-action mapping continues to affect behavior even after as many as 200 trials to a virtually unchanged degree (Experiments 1A,B in Elsner and Hommel, 2001; Experiments 1 and 3 in Herwig and Waszak, 2009). To be clear, we embrace the possibility that the strength of action effect associations or their expression might be boosted when actions are chosen in order to achieve a certain effect (but see Pfister and Kunde, 2013 for the opposite pattern). However, we would like to emphasize that this is not restricted to stimulus-free free-choice cases. For instance, the instruction to freely choose a response when encountering ambiguous stimuli gave rise to a stronger or longer lasting impact of action effects on behavior also when responding to less ambiguous stimuli according to an S–R rule (Gaschler and Nattkemper, 2012). Moreover, we have recently shown that overlearned response-effects are anticipated and thus affect responses to stimuli whenever the participants intend to achieve a specific effect (Zwosta et al., 2013). It might be interesting to explore whether similar short-term and longer-term temporal dynamics apply to rapid action effect learning under free-choice conditions.

## Conflict of Interest Statement

The authors declare that the research was conducted in the absence of any commercial or financial relationships that could be construed as a potential conflict of interest.

## Footnotes

1 A different aspect of part of the data (25 participants in the partly guided group) has been used for cross-validation of the main experiment’s finding reported in Ruge et al. (2012). In particular, the paper focused on the detection of early markers of action effect learning in instruction-based and trial-and-error learning. The analyses capitalized on a correlation of response slowing during learning and the size of the compatibility effect in the test-phase. Importantly, effects of response selection difficulty during acquisition or test were not analyzed. Data was collapsed across experiments with spatial and non-spatial response cues, and only test trials without response cues entered the analysis.

2 Because accuracy was naturally closer to ceiling in trials with response cues, potential differences between compatible and incompatible trials might have been harder to detect, giving rise to a spurious interaction. However, in three additional generalized mixed model analyses on transformed measures of accuracy (d’, logit proportion correct, frequency of accurate responses) the interaction between compatibility and response selection difficulty during test remained significant (Log-linear d’ *F*(1,192) = 5.50, *p* = 0.020, Gamma regression logit proportion correct, *F*(1,192) = 5.22, *p* = 0.023, Log-linear frequency *F*(1,192) = 5.53, *p* = 0.020), which increases our confidence in the interaction effect.

3 A different aspect of part of the data (*N* = 20) has been used for cross-validation of the main experiment’s finding reported in Ruge et al. (2012). In this previous paper effects of response selection difficulty during learning or test were not analyzed. Data was collapsed across experiments with spatial and non-spatial response cues, and only test trials without response cues entered the analysis. Here we extended the initial sample to match the sample size of the groups in Experiment 1.
